# Prevalence and Predictors of Long Covid in a Cohort of Brazilian Adults 12 Months After Acute Infection: A Cross‐Sectional Study

**DOI:** 10.1111/hex.70467

**Published:** 2025-11-14

**Authors:** Eduardo Rocha Covre, Carlos Laranjeira, Lígia Carreira, Carla Franciele Höring, Herbert Leopoldo de Freitas Góes, Vanessa Denardi Antoniassi Baldissera, Priscila Garcia Marques, Viviane Camboin Meireles, Maria Fernanda do Prado Tostes, Rosana Rosseto de Oliveira, Marcelle Paiano, Rosella Santoro Ageno, Márcia Moroskoski, Jesús Puente Alcaraz, João Nickenig Vissoci, Luiz Augusto Facchini, Maria Aparecida Salci

**Affiliations:** ^1^ Department of Postgraduate Nursing, Avenida Colombo State University of Maringá Maringá Brazil; ^2^ School of Health Sciences Portugal; ^3^ Centre for Innovative Care and Health Technology (ciTechCare) Polytechnic University of Leiria Leiria Portugal; ^4^ Comprehensive Health Research Centre (CHRC) University of Évora Évora Portugal; ^5^ Department of Statistics State University of Maringá Maringá Brazil; ^6^ Department of Physical Education State University of Maringá Paraná Brazil; ^7^ Department of Nursing University of Antofagasta Antofagasta Chile; ^8^ Department of Nursing University of Burgos Burgos Spain; ^9^ Division of Emergency Medicine, Duke Global Health Institute Duke University Durham North Carolina USA; ^10^ Department of Postgraduate Studies in Epidemiology, Nursing and Family Health Federal University of Pelotas Pelotas Rio Grande do Sul Brazil

**Keywords:** adults, Brazil, Covid‐19, long Covid, predictors, prevalence

## Abstract

**Introduction:**

Since the onset of the pandemic in early 2020, various reports have emerged regarding persistent symptoms associated with Covid‐19. Nevertheless, there is insufficient data on the persistence of symptoms over time. This study sought to estimate the prevalence of persistent symptoms 12 months after Covid‐19 infection and identify predictors of long Covid in adults living in the State of Paraná, southern Brazil, according to the level of severity of Covid‐19 infection.

**Method:**

An observational and cross‐sectional survey was conducted with Brazilian adults diagnosed with Covid‐19, as assessed from data available in two official Covid‐19 notification databases in Brazil, using telephone interviews. Descriptive statistics, tests of associations and simple and multiple binary logistic regression analysis were used to identify predictors of long Covid.

**Results:**

In total, 1033 adults participated in the study. The overall prevalence of long Covid was 60.3% (*n* = 623). Prevalence was higher in women (67.7%), people aged between 50 and 59 years (65.8%) and in individuals who received treatment in an Intensive Care Unit (ICU) during the acute phase of Covid‐19 infection (74.4%, *n* = 241). The risk factors associated with a greater chance of developing long Covid were: female (OR 2.38; 95% CI 1.55; 3.66), living in the Brazilian northwest health macro‐region (OR 2.20; 95% CI 1.21; 4.00), presenting multimorbidity (OR 1.86; 95% CI 1.06; 3.28), having an average of six symptoms in the acute phase of Covid‐19 (OR 1.22; 95% CI 1.17; 1.28) and having received treatment in an ICU (OR 4.86; 95% CI 2.83; 8.35) and inpatient ward (OR 2.45; 95% CI 1.47; 4.09).

**Conclusions:**

The results highlight the high prevalence of long Covid and support the formulation of health policies capable of minimising the consequences on the population, on the services offered by professionals and on health systems.

**Patient or Public Contribution:**

The study topic's importance was based on the patients' experiences in the author's previous research and the need to develop patient‐centred care.

## 1 Introduction

Four years after the global Covid‐19 pandemic, the scientific community continues to investigate long‐term morbidity associated with the severe acute respiratory syndrome coronavirus 2 (SARS‐CoV‐2) infection. As SARS‐CoV‐2 progressed from the wild‐type strain to the Alpha, Beta, Delta and Omicron variants, there may be a variant‐specific impact on long Covid similar to that of the acute disease [1]. Despite the emergence of new Covid‐19 strains and variants, since the first wave of infection in 2020 and the advent of vaccination, people affected by the disease have often reported the persistence or recurrence of symptoms months after its acute phase. This condition is described as post‐acute Covid or long Covid, where Covid‐19 symptoms last for 3–12 weeks or more than 12 weeks, respectively [2, 3, 4, 5]. An estimated 65 million individuals globally are afflicted by long Covid, with incidence rising daily [6]. The incidence is estimated at 50%–70% of hospitalised cases, 10%–30% of non‐hospitalised cases and 10%–12% of vaccinated cases [7, 8].

Long Covid is a multisystem disorder characterised by simultaneous symptomatologic manifestations, such as tiredness/fatigue, shortness of breath, cough, pain in muscles and joints, anxiety, depression, and changes in smell and taste, among others [9, 10, 11, 12]. Its late symptoms affect people's lives negatively, preventing them from re‐establishing their health conditions before Covid‐19 and, consequently, impacting their quality of life and interfering with the performance of daily living activities and professional and social activities [13, 14, 15].

Due to the topic's contemporaneity, the global scientific community has made unprecedented efforts to describe long Covid and identify the factors related to its development. The literature reveals wide variations in occurrence and prevalence rates among adults, as well as substantial differences between those who have had mild, moderate and severe cases of Covid‐19 [16, 17, 18, 19].

Higher rates of long Covid were evident in more severe patients, that is, those who required hospitalisation [18, 20, 21]. Prevalence rates were also associated with socio‐demographic characteristics, health conditions before the infection, viral load and its persistence in the body, systemic sequelae, manifestation and duration of symptoms and severity of the disease in the acute phase [22, 23, 24, 25].

Families, health systems and society in general have been overwhelmed in their efforts to deal with, resolve or minimise the problems triggered by long Covid [11]. Given this scenario and the topic's novelty, it becomes extremely relevant to evaluate the predictors of long Covid in different populations, mainly in adults (as they are professionally and economically active), and to understand this condition's impact on these people's lives to promote health and guarantee health protection, recovery and rehabilitation.

In this sense, this study aims to estimate the prevalence of persistent symptoms 12 months after Covid‐19 infection and identify predictors of long Covid in adults living in the State of Paraná, southern Brazil, according to the level of severity of Covid‐19 infection.

## 2 Materials and Methods

### 2.1 Study Design

This observational cross‐sectional study is part of a larger project using data from a Brazilian cohort of adults and older people called ‘Cohort COVID‐19 Paraná/UEM’ [26]. The study follows the Strengthening the Reporting of Observational Studies in Epidemiology (STROBE) checklist [27].

### 2.2 Setting, Participants and Recruitment

The study was carried out in the state of Paraná, located in southern Brazil. This state has 11,443,208 inhabitants [28] and 399 municipalities, grouped into 22 health regions across four macro‐regions (Northwest, North, West and East) [29].

The cohort includes adults aged between 18 and 59 years, diagnosed with Covid‐19, through laboratory confirmation (nasopharyngeal swab RT‐qPCR), from 11 March to 31 December 2020. Before 7 June 2021, the alpha variant was dominant in the population [30]. Inclusion in the study required that participants reside in and had undergone treatment for acute Covid‐19 infection in the State of Paraná. Participants were evaluated 12 months after Covid‐19 infection and grouped according to disease severity and place of treatment during the acute phase: mild cases (outpatients who did not require hospitalisation); moderate cases (patients who were hospitalised in a ward) and severe/critical cases (patients who required intensive care in Intensive Care Units (ICUs) or those who underwent invasive mechanical ventilation) [26].

### 2.3 Data Collection

Considering treatment as an indicator of the acute disease's severity, the initial selection of potential cohort participants was carried out using two official Covid‐19 notification databases in Brazil. Outpatient (mild) cases were obtained from the official database of the State of Paraná, entitled ‘NOTIFICA COVID‐19 PARANÁ’, while moderate and severe cases were obtained from the official database of the country's Ministry of Health, SIVEP‐Influenza (Influenza Epidemiological Surveillance Information).

To select the sample, a proportional stratified probabilistic approach was used, considering the health macro‐region of the individual's municipality of residence and the month they were reported with the disease. The sample size was defined using the Feiss continuity correction method [31], considering a 95% confidence interval, 5% significance and 80% statistical power. At the same time, a dropout rate of 20% and a post‐discharge death rate from Covid‐19 of 25% were estimated. The calculations indicated a sample size of at least 900 participants.

### 2.4 Data Collection

Data collection was carried out through telephone contacts, using a specific electronic form prepared by the researchers, composed of questions related to the object of study and covering socio‐demographic characteristics, lifestyle, clinical and treatment information. Before data collection, interviewers were trained, and a pilot study was conducted with the aim of standardising research procedures. Data collection took place from March to December 2021 (12 months after acute Covid‐19 infection).

### 2.5 Study Variables

#### 2.5.1 Independent Variables

I. Socio‐demographic characteristics: sex (male and female); age group (19–29, 30–39, 40–49 and 50–59 years); race (non‐white and white); years of education (up to 8 years of study and more than 8 years of study); family income, in Brazilian minimum wages, estimated per capita (up to 1 minimum wage, 1–2 minimum wages, 2–3 minimum wages and more than 3 minimum wages); and health macro‐region (East, West, North and Northwest).
II. Health variables pre‐Covid‐19: alcohol use (yes/no); tobacco use (yes/no); physical activity (yes/no); having comorbidities (none, 1, 2 or more); long‐term medication use (yes/no); number of symptoms in the acute phase of Covid‐19 (none, 1, 2, 3 or more); nutritional status (underweight, normal weight, overweight and obesity). Nutritional status was classified for adults (up to 59 years old), according to the Ministry of Health [32]: underweight (≤ 18.5 kg/m^2^); eutrophic (≥ 18.5–24.9 kg/m^2^), overweight (≥ 25.0 and < 29.9 kg/m^2^) and obesity (≥ 30.0 kg/m^2^).
III. Management and treatment of acute Covid‐19 infection: use of ventilatory support (yes/no; if yes, invasive or non‐invasive); treatment by SUS (Unified Health System) [Public Healthcare System] (yes/no); monitoring by UBS (Basic Health Unit) (yes/no); severity of acute Covid‐19 infection (mild, moderate and severe/critical). According to the World Health Organization [33], the severity of Covid‐19 symptoms is classified as (1) Mild Cases: asymptomatic individuals or those exhibiting non‐specific symptoms of viral pneumonia; (2) Moderate Cases: mild indicators of viral pneumonia, including SpO2 ≥ 90% on ambient air; (3) Severe/Critical Cases: presence of indicators of severe viral pneumonia alongside a respiratory rate ≥ 30 breaths per minute, dyspnoea or SpO2 ≤ 90% on ambient air; or continuation of severe pneumonia symptoms for over a week, onset of SARS, sepsis, septic shock, acute thrombosis or Multisystem Inflammatory Syndrome.

#### 2.5.2 Outcome Variable

The outcome of the study was the presence of long Covid (yes/no). This is defined as the persistence or recurrence of one or more symptoms of Covid‐19, 12 weeks or more after its acute phase [34, 35]. In total, 33 symptoms were catalogued and grouped according to their related system: neurological (headache, pain in the eyes, change in vision, change in smell, change in taste, change in speech, change in hearing, ringing in the ear, tingling or numbness throughout the body, dizziness, loss of movement co‐ordination and memory loss); respiratory (runny nose, sore throat, hoarse voice, cough, phlegm production, chest pain and shortness of breath); gastrointestinal (change in stool, change in appetite, nausea, cramps or abdominal pain); endocrine (hair loss and sweating at rest); dermatological (spots on the body and itching); musculoskeletal (muscle and joint problems and tiredness/fatigue), cardiocirculatory (oedema) and psychological (depression and anxiety).

### 2.6 Statistical Analysis

All data were tabulated in electronic spreadsheets and were later imported into the R software, version 4.2.0 [36], for descriptive and inferential analysis. Descriptive statistics of the data included absolute and relative frequencies, mean, standard deviation and median. To verify the association between the outcome variable and the explanatory variables of interest, the *χ* ^2^ or Fisher's exact test was performed, when necessary, at a 5% level of significance. Simple and multiple binary logistic regression analysis was used to identify factors associated with long Covid. Univariate logistic regression models and the complete model with all covariates were adjusted. All baseline variables with a *p* value < 0.20 in univariable analysis were recruited for the final model. From the complete model, the stepwise method was applied to select variables and adjust the final model. The adequacy of this model was verified with the analysis of randomised quantile residuals. Collinearity was tested with the Variance Inflation Factor (VIF). Associations were estimated using crude and adjusted odds ratios (ORs) with a 95% confidence interval. Missing data were not imputed and were excluded from the analysis.

### 2.7 Ethics

Ethical approval was granted by the State University of Maringá, under opinion number: 4165272 and CAAE: 34787020.0.0000.0104 on 21 July 2020, and Hospital do Trabalhador, which responds to the Health Department of the State of Paraná, under number opinion: 4214589 and CAAE: 34787020.0.3001.5225 on 15 August 2020. Participants verbally consented to the research, their consent was registered, and the informed consent term was sent by post or email.

## 3 Results

### 3.1 Participants Characterisation

The study participants included 1033 adults diagnosed with Covid‐19 in the State of Paraná, Brazil. The overall prevalence of long Covid among participants was 60.3% (*n* = 623). Prevalence was higher among female participants (*n* = 319; 67.7%), those aged between 50 and 59 years (*n* = 212; 65.8%), those with two or more comorbidities (*n* = 132; 78.1%), those with three or more symptoms in the acute phase of Covid‐19 infection (*n* = 468; 69%), those who received invasive ventilatory support (*n* = 85; 86.7%) and those treated in the public health system (*n* = 334; 63.5%). Furthermore, prevalence increased proportionally according to the severity of the acute Covid‐19 infection, with higher rates in adults who received treatment in the ICU (*n* = 241; 74.4%), followed by those who were treated in the inpatient ward (*n* = 198; 67.3%) and as outpatients (*n* = 184; 44.3%), as described in Table 1.

**Table 1:** Prevalence of long Covid and association with socio‐demographic, health and treatment characteristics according to the severity of Covid‐19 infection. Paraná, Brazil (*N* = 1033).

Variables	Total *N* = 1033		Long Covid								*p* value*
			Total (*n* = 623; 60.3%)		Ambulatory (*n* = 415) (*n* = 184; 44.3%)		Inpatient ward (*n* = 294) (*n* = 198; 67.3%)		ICU (*n* = 324) (*n* = 241; 74.4%)		
	*n*	%	*n*	%	*n*	%	*n*	%	*n*	%	
**Socio‐Demographic Variables**											
Sex											**< 0.001**
Male	562	54.4	304	54.1	59	31.6	105	61.0	140	69.0	
Female	471	45.6	319	67.7	125	54.8	93	76.2	101	83.5	
Age group (years)											**< 0.001**
18–29	175	16.9	86	49.1	55	41.0	20	83.3	11	64.7	
30–39	235	22.7	132	56.2	49	43.8	36	65.5	47	69.1	
40–49	301	29.1	193	64.1	48	50.5	67	63.8	78	77.2	
50–59	322	31.2	212	65.8	32	43.2	75	68.2	105	76.1	
Ethnicity											**0.030**
Non‐white	249	24.1	161	64.7	57	47.1	44	77.2	60	84.5	
White	462	44.7	310	67.1	79	50.3	117	74.5	114	77.0	
Not informed	322	31.2	152	47.2	48	35.0	37	46.3	67	63.8	
Education (years of study)											**0.001**
≤ 8	113	10.9	80	70.8	13	39.4	25	78.1	42	87.5	
> 8	581	56.2	378	65.1	122	50.2	131	74.4	125	77.2	
Not informed	339	32.8	165	48.7	49	35.3	42	48.8	74	64.9	
Family income^a^											0.818
< 1 minimum wage	147	14.2	111	75.5	31	58.5	35	76.1	45	93.8	
1–2 minimum wage	113	10.9	88	77.9	24	68.6	28	80.0	36	83.7	
2–3 minimum wage	44	4.3	33	75.0	12	75.0	11	78.6	10	71.4	
> 3 minimum wage	60	5.8	43	71.7	13	65.0	10	66.7	20	80.0	
Not informed	669	64.8	348	52.0	104	35.7	114	62.0	130	67.0	
Health macro‐region											**< 0.001**
East	499	48.3	308	61.7	61	41.5	91	63.6	156	74.6	
West	241	23.3	138	57.3	64	47.1	36	62.1	38	80.9	
North	129	12.5	71	55.0	25	39.1	23	76.7	23	65.7	
Northwest	164	15.9	106	64.6	34	50.0	48	76.2	24	72.7	
**Health Variables Pre‐Covid‐19**											
Alcohol use											0.821
No	453	43.9	306	67.5	87	50.0	101	77.7	118	79.2	
Yes	338	32.7	220	65.1	66	48.5	75	72.1	79	80.6	
Not informed	242	23.4	97	40.1	31	29.5	22	36.7	44	57.1	
Tobacco use											**0.017**
No	758	73.4	502	66.2	136	47.9	169	74.8	197	79.4	
Yes	46	4.5	30	65.2	15	53.6	9	90.0	6	75.0	
Not informed	229	22.2	91	39.7	33	32.0	20	34.5	38	55.9	
Physical activity											0.764
No	364	35.2	227	62.4	67	48.6	75	68.2	85	73.3	
Yes	424	41.0	290	68.4	79	47.6	104	80.6	107	82.9	
Not informed	245	23.7	106	43.3	38	34.2	19	34.5	49	62.0	
Comorbidity											**< 0.001**
None	588	56.9	308	52.4	122	41.5	91	61.5	95	65.1	
1	276	26.7	183	66.3	40	44.9	63	74.1	80	78.4	
> 2	169	16.4	132	78.1	22	68.8	44	72.1	66	86.8	
Long‐term medication											**< 0.001**
No	390	37.8	238	61.0	95	48.7	64	70.3	79	76.0	
Yes	406	39.3	291	71.7	59	50.0	107	75.9	125	85.0	
Not informed	237	22.9	94	39.7	30	29.4	27	43.5	37	50.7	
Body Mass Index											**< 0.001**
Underweight/Eutrophic	149	14.4	80	53.7	48	45.3	19	73.1	13	76.5	
Overweight	246	23.8	162	65.9	52	53.6	56	69.1	54	79.4	
Obesity	288	27.9	205	71.2	32	46.4	74	75.5	99	81.8	
Not informed	350	33.9	176	50.3	52	36.4	49	55.1	75	63.6	
Covid‐19 symptoms											**0.022**
0	195	18.9	90	46.2	23	31.1	23	45.1	44	62.9	
1	74	7.2	30	40.5	7	19.4	7	41.2	16	76.2	
2	86	8.3	35	40.7	17	32.1	7	43.8	11	64.7	
> 3	678	65.6	468	69.0	137	54.4	161	76.7	170	78.7	
**Treatment and Management of Acute Covid‐19 Infection**											
Use of ventilatory support											**< 0.001**
No	482	46.7	243	50.4	163	44.9	57	64.8	23	74.2	
Yes. Not invasive	283	27.4	211	74.6	7	87.5	107	75.4	97	72.9	
Yes. Invasive	98	9.5	85	86.7	1	50.0	7	100.0	77	86.5	
Not informed	170	16.5	84	49.4	13	31.0	27	47.4	44	62.0	
Treatment by SUS											**0.046**
No	507	49.1	289	57.0	72	41.9	102	61.1	115	68.5	
Yes	526	50.9	334	63.5	112	46.1	96	75.6	126	80.8	
Monitoring by UBS											0.056
No	507	49.1	315	62.1	83	42.8	113	72.9	119	75.3	
Yes	254	24.6	181	71.3	66	55.5	51	79.7	64	90.1	
Not informed	272	26.3	127	46.7	35	34.3	34	45.3	58	61.1	

Abbreviations: ICU, Intensive Care Unit; SUS, Unified Health System; UBS, Basic Health Unit.

^a^ In 2021, the minimum wage in Brazil was R$1100.00.

* Pearson's *χ* ^2^ test; Fisher's exact test. The category called ‘Not informed’ was not considered in the associations. Values in bold are statistically significant (*p* < 0.05).

When comparing the three levels of severity of acute Covid‐19 infection, females with more severe acute cases were more likely to present prolonged symptoms of the disease (ICU: 83.5%, inpatient ward: 76.2% and ambulatory: 54.8%). In adults aged between 40 and 49 years, persistent symptoms were more prevalent among those who received ambulatory treatment (50.5%) and ICU (77.2%), and in younger adults (18–29 years old), among those who received treatment in an inpatient ward (83.3%).

Overall, and when comparing Covid‐19 severity levels, there was a higher prevalence of persistent symptoms of long Covid in adults who reported two or more comorbidities (ambulatory: 68.8% and ICU: 86.8%). Among those treated in the inpatient ward, the predominance was 74.1% in people with just one comorbidity. The prevalence of long Covid was greatest among those with more severe cases and with a higher number of symptoms in the acute phase. Therefore, in adults who reported three or more acute symptoms of Covid‐19, there was a predominance of persistent symptoms (69.0%) in the three exposure groups (ambulatory: 54.4%; inpatient ward: 76.7% and ICU: 78.7%).

In participants who required invasive ventilatory support and treatment by the SUS, the prevalence of long Covid increased with greater severity of Covid‐19, that is, there was a greater predominance of prolonged symptoms of long Covid in people who were hospitalised in inpatient ward (100%) and ICU (86.5%) and who were treated by the public health system (ICU: 80.8%; inpatient ward: 75.6% and ambulatory: 46.1%).

### 3.2 Prevalence of Symptoms Related to Long Covid

The most highly prevalent persistent symptoms were related to neurological symptoms (52.2%; *n* = 539), followed by musculoskeletal (39.7%; *n* = 410) and respiratory symptoms (34.6%; *n* = 357), as described in Table 2.

**Table 2:** Prevalence of symptoms related to long Covid according to the severity of acute Covid‐19 infection. Paraná, Brazil (*N* = 1033).

Symptoms	Long Covid								*p* value*
	Total (*n* = 623; 60.3%)		Ambulatory (*n* = 415) (*n* = 184; 44.3%)		Inpatient ward (*n* = 294) (*n* = 198; 67.3%)		ICU (*n* = 324) (*n* = 241; 74.4%)		
	*n*	%	*n*	%	*n*	%	*n*	%	
Neurological	539	52.2	157	37.8	165	56.1	217	67.0	0.104
Headache	151	14.6	48	11.6	54	18.4	49	15.1	0.188
Eye pain	55	5.3	15	3.6	20	6.8	20	6.2	0.745
Change in vision	127	12.3	25	6.0	39	13.3	63	19.4	**0.006**
Change in smell	170	16.5	67	16.1	49	16.7	54	16.7	**0.003**
Change in taste	157	15.2	59	14.2	44	15.0	54	16.7	**0.038**
Change in speech	54	5.2	9	2.2	14	4.8	31	9.6	**0.009**
Change in hearing	35	3.4	11	2.7	11	3.7	13	4.0	0.966
Ringing in the ear	44	4.3	13	3.1	14	4.8	17	5.2	0.999
Tingling/numbness throughout the body	106	10.3	16	3.9	25	8.5	65	20.1	**< 0.001**
Dizziness	102	9.9	20	4.8	32	10.9	50	15.4	**0.024**
Loss of co‐ordination of movements	100	9.7	10	2.4	17	5.8	73	22.5	**< 0.001**
Memory loss	384	37.2	104	25.1	126	42.9	154	47.5	0.235
Respiratory	357	34.6	84	20.2	119	40.5	154	47.5	**0.005**
Runny nose	29	2.8	5	1.2	8	2.7	16	4.9	0.145
Sore throat	45	4.4	17	4.1	14	4.8	14	4.3	0.398
Hoarse voice	60	5.8	14	3.4	13	4.4	33	10.2	**0.022**
Cough	136	13.2	27	6.5	52	17.7	57	17.6	**0.016**
Phlegm production	43	4.2	9	2.2	14	4.8	20	6.2	0.387
Chest pain	108	10.5	23	5.5	39	13.3	46	14.2	0.117
Shortness of breath	251	24.3	54	13.0	85	28.9	112	34.6	**0.001**
Gastrodigestive	182	17.6	37	8.9	67	22.8	78	24.1	**0.005**
Change in stool	62	6.0	9	2.2	26	8.8	27	8.3	**0.019**
Change in appetite	130	12.6	29	7.0	47	16.0	54	16.7	0.120
Nausea	29	2.8	5	1.2	12	4.1	12	3.7	0.287
Abdominal cramps/pain	26	2.5	5	1.2	11	3.7	10	3.1	0.382
Endocrine	289	28.0	86	20.7	98	33.3	105	32.4	0.461
Hair loss	246	23.8	78	18.8	78	26.5	90	27.8	0.573
Perspiration at rest	86	8.3	18	4.3	37	12.6	31	9.6	**0.036**
Skin	54	5.2	3	0.7	20	6.8	31	9.6	**0.002**
Stains on the body	34	3.3	3	0.7	15	5.1	16	4.9	**0.022**
Pruritus	34	3.3	2	0.5	12	4.1	20	6.2	**0.004**
Musculoskeletal	410	39.7	103	24.8	139	47.3	168	51.9	**0.003**
Muscle/joint problems	132	12.8	28	6.7	43	14.6	61	18.8	**0.040**
Tiredness/fatigue	384	37.2	93	22.4	133	45.2	158	48.8	**0.001**
Cardiovascular	86	8.3	18	4.3	37	12.6	31	9.6	**0.036**
Oedema	61	5.9	12	2.9	14	4.8	35	10.8	**0.006**
Psychological	244	23.6	64	15.4	79	26.9	101	31.2	0.318
Depression	174	16.8	44	10.6	57	19.4	73	22.5	0.330
Anxiety	192	18.6	54	13.0	65	22.1	73	22.5	0.743

Abbreviation: ICU, Intensive Care Unit.

* Pearson's *χ* ^2^ test; Fisher's exact test; Values in bold are statistically significant (*p* < 0.05).

Long Covid was associated with the following neurological symptoms: changes in vision, changes in smell, changes in taste, changes in speech, tingling or numbness throughout the body, dizziness and loss of co‐ordination of movements. Notably, all symptoms increase in prevalence with increased severity of Covid‐19; that is, these symptoms were more evident in individuals who were treated in the ICU, always at rates above the global prevalence. Symptoms such as headache and memory loss, although they did not show statistically significant associations, had a high prevalence, 14.6% (*n* = 151) and 37.2% (*n* = 384), respectively.

Long Covid was associated with the following respiratory symptoms: hoarse voice, cough and shortness of breath were significant. Musculoskeletal symptoms included problems with muscles/joints and tiredness/fatigue. Shortness of breath (24.3%; *n* = 251) and tiredness/fatigue (37.2%; *n* = 384) were the most prevalent symptoms at all severity levels of Covid‐19 infection, and their prevalence increased proportionally to the severity of Covid‐19, with the highest prevalence among individuals treated in the ICU.

### 3.3 Factors Associated With Self‐Reported Long Covid

Table 3 presents the crude and adjusted logistic regression models for the associations between socio‐demographic variables, health variables before Covid‐19, and variables related to the management and treatment of Covid‐19 in study participants. Among the socio‐demographic characteristics, gender and health macro‐region of residence were significantly associated with long Covid (*p* < 0.005). Females (*p* < 0.001) were more than twice as likely (OR 2.38; 95% CI 1.55–3.66) of having long Covid as males. Regionally, residents of the Northwest (*p* = 0.009) were 79% more likely to develop long Covid (OR 2.20; 95% CI 1.21; 4.00), when compared to residents of the East.

**Table 3:** Crude and adjusted logistic regression analysis to estimate the association between the participants' characteristics and the presence of persistent symptoms of long Covid. Paraná, Brazil (*N* = 623).

Variables	Long Covid		Crude model			Adjusted model		
	*n*	%	Estimates	OR (95% CI)	*p* value	Estimates	aOR (95% CI)	*p* value
**Socio‐Demographic Variables**								
Sex								
Male	304	48.8	Reference	—	—	Reference	—	—
Female	319	51.2	0.57	1.78 (1.38–2.30)	**< 0.001**	0.86	2.38 (1.55–3.66)	**< 0.001**
Age group (years)								
18–29	86	13.8	Reference	—	—	Reference	—	—
30–39	132	21.2	0.28	1.33 (0.9–1.96)	0.158	—	—	—
40–49	193	31.0	0.61	1.85 (1.27–2.70)	**0.001**	0.74	1.56 (0.92–2.64)	0.101
50–59	212	34.0	0.69	1.99 (1.37–2.90)	**0.003**	0.79	1.64 (0.87–3.10)	0.129
Race								
Non‐White	161	34.2	Reference	—	—	—	—	—
White	310	65.8	0.10	1.11 (0.81–1.54)	0.511	—	—	—
Education (study years)								
≤ 8	80	17.5	Reference	—	—	—	—	—
> 8	378	82.5	−0.26	0.77 (0.49–1.19)	0.239	—	—	—
Family income^a^								
< 1 minimum wage	111	40.4	Reference	—	—	—	—	—
1–2 minimum wage	88	32.0	0.13	1.14 (0.64–2.04)	0.655	—	—	—
2–3 minimum wage	33	12.0	−0.02	0.97 (0.45–2.12)	0.945	—	—	—
> 3 minimum wage	43	15.6	−0.19	0.82 (0.42–1.61)	0.565	—	—	—
Health macro‐region								
East	308	49.4	Reference	—	—	Reference	—	—
West	138	22.2	−0.18	0.83 (0.61–1.14)	0.245	—	—	—
North	71	11.4	−0.27	0.76 (0.51–1.12)	0.167	—	—	—
Northwest	106	17.0	0.12	1.13 (0.78–1.64)	**0.031**	0.79	2.20 (1.21–4.00)	**0.009**
**Health Variables Pre‐Covid‐19**								
Alcohol use								
No	306	58.2	Reference	—	—	—	—	—
Yes	220	41.8	−0.11	0.9 (0.66– 1.21)	0.468	—	—	—
Tobacco use								
No	502	94.4	Reference	—	—	—	—	—
Yes	30	5.6	−0.04	0.96 (0.51–1.79)	0.888	—	—	—
Physical activity								
No	227	43.9	Reference	—	—	—	—	—
Yes	290	56.1	−0.26	0.77 (0.57–1.03)	0.075	—	—	—
Comorbidity								
0	308	49.4	Reference	—	—	Reference	—	—
1	183	29.4	0.58	1.79 (1.33–2.41)	**0.001**	0.28	1.33 (0.83–2.13)	0.236
> 2	132	21.2	1.17	3.24 (2.18–4.83)	**< 0.001**	0.86	1.86 (1.06–3.28)	**0.031**
Long‐term medication								
No	238	45.0	Reference	—	—	—	—	—
Yes	291	55.0	0.48	1.62 (1.2–2.17)	0.081	—	—	—
Body Mass Index								
Underweight/Eutrophic	80	17.9	Reference	—	—	Reference	—	—
Overweight	162	36.2	0.50	1.66 (1.10–2.52)	0.116	—	—	—
Obesity	205	45.9	0.75	2.13 (1.41–3.21)	**0.003**	1.02	1.63 (1.40–1.21)	0.394
Covid‐19 symptoms	MD = 6; SD = 6.17		0.16	1.17 (1.14–1.20)	**< 0.001**	0.20	1.22 (1.17–1.28)	**< 0.001**
**Management and Treatment of Acute Covid‐19 Infection**								
Severity of acute Covid‐19 infection								
Mild cases (Ambulatory)	184	29.5	Reference	—	—	Reference	—	—
Moderate cases (Inpatient Ward)	198	31.8	0.95	2.59 (1.90–3.54)	**< 0.001**	1.90	2.45 (1.47–4.09)	**0.006**
Severe/critical cases (ICU)	241	38.7	1.29	3.65 (2.66–5.00)	**< 0.001**	1.58	4.86 (2.83–8.35)	**< 0.001**
Ventilatory support use								
No	243	45.1	Reference	—	—	Reference	—	—
Yes. Not invasive	211	39.1	1.05	2.88 (2.09–3.98)	**< 0.001**	1.36	1.14 (0.81–2.09)	0.092
Yes. Invasive	85	15.8	1.86	6.43 (3.49–11.84)	**< 0.001**	1.94	3.68 (2.13–5.53)	0.236
Treatment by SUS								
No	289	46.4	Reference	—	—	Reference	—	—
Yes	334	53.6	0.27	1.31 (1.02–1.68)	**0.033**	−0.53	0.59 (0.38–0.91)	**0.016**
Monitoring by UBS								
No	315	63.5	Reference	—	—	Reference	—	—
Yes	181	36.5	0.41	1.51 (1.09–2.09)	**0.013**	0.39	1.48 (0.95–2.30)	0.082

*Note:* The category labelled ‘not informed’ was not considered in the associations. Values in bold are statistically significant (*p* < 0.05).

Abbreviations: aOR, adjusted odds ratio; CI, confidence interval; ICU, Intensive Care Unit; M, mean; MD, median; OR, odds ratio; SD, standard deviation; SUS, Unified Health System; UBS, Basic Health Unit.

^a^ In 2021, the minimum wage in Brazil was R$1100.00.

Among health variables, multimorbidity and having several acute symptoms of Covid‐19 were associated with long Covid. Adults with two or more comorbidities (*p* = 0.031) were 86% more likely (OR 1.86; 95% CI 1.06; 3.28) to display persistent symptoms, when compared to those without comorbidities. Those with a median of six acute symptoms of Covid‐19 (*p* < 0.001) were 20% more likely (OR 1.22; 95% CI 1.17; 1.28) of developing long Covid, when compared to asymptomatic individuals.

The variables related to the management and treatment of Covid‐19 were significantly associated with long Covid in participants who were treated in an inpatient ward (*p* = 0.006) and the ICU (*p* < 0.001). When compared to participants treated on an ambulatory basis, adults hospitalised in an inpatient ward (OR 2.45; 95% CI 1.47; 4.09) and ICU (OR 4.86; 95% CI 2.83; 8.35) were two times and nearly five times as likely to develop long Covid, respectively. Receiving treatment through the SUS was identified as a protective factor against long Covid, that is, it reduced the chance of presenting persistent symptoms by 41% (OR 0.59; 95% CI 0.38; 0.91), when compared to those who did not use the system (Table 3).

## 4 Discussion

As one of the consequences of the Covid‐19 pandemic, long Covid emerges as a global public health problem and has become one of the main objects of research in recent years [37]. In this study, the prevalence of long Covid and its associated factors were estimated in adults living in the State of Paraná, southern Brazil. To this end, a cross‐sectional analysis was carried out using data from a cohort in which participants were retrospectively assessed 12 months after the date of discharge from where they received treatment for the acute phase of Covid‐19 [26].

Comparing recent studies conducted in different contexts, there is a wide variation in the occurrence and prevalence of long Covid in people aged between 18 and 59 years. An estimated 3.4%–93% of adults may have this condition [22, 23, 25, 38]. These disparities can be justified by several factors, such as: (a) study designs and target populations; (b) the duration and type of follow‐up; (c) the temporal and diagnostic criteria used to define long Covid; (d) the list of symptoms analysed; and finally, (e) the capacity and accuracy of health systems in diagnosis and initial notification of Covid‐19 [39, 40, 41]. More recently, a meta‐analysis reported a global pooled long Covid prevalence of 36%, with the highest prevalence rate observed in South America (51%) [42]. Different studies from Brazil state that the prevalence of long Covid is over 60% at 12 months [18, 43]. While the prevalence of long Covid may vary over time, Hou et al. [42] also stated a high burden of symptoms 1–2 years post‐infection.

Given the topic's novelty, the duration and underlying mechanism(s) of long Covid are still uncertain and inconclusive. Hypotheses have emerged suggesting that the persistence, recurrence and/or development of Covid‐19 symptoms over time are closely related to an autoimmune response [44] and mast cell activation [45], the persistence of SARS‐CoV‐2 in the body [46] or dysregulated inflammatory response [46, 47, 48]. Therefore, studies that define the risk factors for the occurrence of long Covid are of utmost importance, as they contribute towards our understanding of the problem.

Different risk factors associated with greater chances of symptom persistence have been identified in the literature, such as increasing age, active smoking, overweight and obesity, pre‐existing lung disorders and chronic diseases [49, 50]. Additionally, this study highlights the female sex, the presence of two or more underlying diseases, the manifestation of three or more symptoms in the acute phase of Covid‐19 and hospital treatment (in an inpatient ward and/or ICU).

Sex is considered a significant factor in the outcome of several diseases and has already been associated with outcomes and sequelae caused by the coronavirus [51, 52]. At the beginning of the Covid‐19 pandemic, men were more affected by the disease than women, showing higher rates of hospital admission (mainly in the ICU), more unfavourable outcomes and, consequently, higher rates of lethality and mortality [53, 54]. However, with the development of long Covid, this scenario has changed, and the male sex seems to exhibit a protective factor for the development of prolonged Covid‐19 symptoms [55]. Conversely, the prevalence and probability of long Covid are significantly higher among women [25, 38, 56, 57, 58, 59, 60]. In this study, when compared to men, women are twice as likely to develop persistent symptoms of Covid‐19.

Assuming that long Covid is due to an autoimmune response, studies suggest that the high prevalence and greater chances of this condition affecting women are due to their faster and more robust adaptive immune responses of IgG antibodies (Immunoglobulin G) in the acute phase of Covid‐19, which protects them against the disease and its worsening. However, this makes them more vulnerable to its manifestations and sequelae [11, 47], as well as any imbalance that occurs in their bodily health [61], although women, in general, are more attentive to their bodies. Additionally, female hormones are assumed to play an important role in perpetuating the hyperinflammatory state of the acute phase of Covid‐19.

Studies have already shown that Covid‐19 patients, regardless of the treatment regimen during its initial phase, have shown prolonged symptoms of the disease [20, 21, 39, 62]. However, patients hospitalised in wards and ICUs are, respectively, three and five times more likely to develop long Covid when compared to those treated in outpatient settings [57, 63, 64]. The results of this study are corroborated by the evidence found in the literature, which reveals that adults who developed more severe forms of Covid‐19, that is, who were hospitalised in wards and ICUs, are two and almost five times more likely to develop long Covid, respectively.

The presence of comorbidities was also identified as a significant predictor of long Covid. The finding is in line with other studies showing an association between the presence of comorbidities and long Covid [41, 62, 65]. Research carried out in the European continent demonstrated that a wide range of comorbidities—such as fibromyalgia, depression, anxiety, COPD, benign prostatic hyperplasia, multiple sclerosis and coeliac disease, among others—are associated with an increased risk of prolonged Covid‐19 symptoms up to 12 weeks after infection [62].

Regarding the symptomatologic manifestations associated with long Covid, a scoping review catalogued more than 100 symptoms and related them to cardiovascular, pulmonary, respiratory, neurological, psychological, sensory, dermatological, functional and gastrointestinal symptoms, among other organs and systems. The prevalence of symptoms varies significantly from 10% to 70%, with respiratory problems (dyspnoea/shortness of breath) reported most often, followed by neurological (olfactory dysfunctions) and musculoskeletal problems (tiredness/fatigue) [65]. In this survey, at least one neurological, respiratory and musculoskeletal symptom was reported by 177 (28.4%), 170 (27.2%) and 304 (48.7%) adults, respectively. Available evidence suggests that these symptoms tend to increase over time [67]. Studies investigating the occurrence of long Covid in adults identified a list of related symptoms, such as: anxiety and depression, tiredness/fatigue, shortness of breath, musculoskeletal changes, difficulty concentrating and memory disorders, cognitive dysfunctions, smell and taste disorders, cough, headache, chest pain, vomiting, recurrent tonsillitis, gastrointestinal changes, hair loss, changes in vision and increased production of secretions in the airways [10, 42, 56, 60, 65, 68, 69].

Long Covid is still a poorly understood multisystem disorder that involves persistent physical and mental sequelae after Covid‐19's acute phase [11, 34]. Given the heterogeneity of symptoms, continuous and thorough observation is required, as well as multidisciplinary stepwise care approaches to identify and improve symptoms.

There is evidence that persistent and/or recurrent symptoms of Covid‐19 improve over time, but some tend to last longer than others [68]. Individuals with chronic symptoms experience a long‐term impact on their health status, whether through increased functional disability and decreased quality of life, or through lost days or unproductiveness at work [70, 71]. Indeed, person‐centred care is needed with the early identification of persistent symptoms based on the main associated risk factors [22, 72].

At the same time, a substantial increase in the use of healthcare services and, consequently, direct medical costs associated with long Covid has been observed. However, a significant percentage of people with long‐lasting Covid‐19 symptoms are expected to recover without needing hospital care. The greatest use of resources and services includes visits to Emergency Care Units (UPA), periodic consultations with Primary Health Care (PHC) professionals, visits to specialists, especially rehabilitation services (physiotherapy, occupational therapy and speech therapy) and physiatry [38, 55, 68, 72, 73, 74].

Therefore, all persistent manifestations after the disease must be monitored by a qualified multidisciplinary team, due to the possibility of changes in the health–disease process and, consequently, in quality of life. To this end, national health strategies are necessary to combat long Covid, such as the development of clinical guidelines and training of professionals to identify, evaluate and care for affected people [75]. To this end, monitoring guidelines were created in Brazil to be followed by the Unified Health System (SUS), through PHC, related to all persistent symptoms documented in the scientific literature. Recommendations include holistic and longitudinal management (carried out through structured visits in PHC) and referrals to specialised multidisciplinary and rehabilitation services, according to the duration of symptoms and training of affected people [76, 77, 78].

The healthcare system in Brazil is made up of private providers (paid by health plans or out of pocket) and public providers (paid directly by the government). The SUS represents the public provider, with universal access, and is the only provider of healthcare for more than 70% of the Brazilian population [79]. However, demand created by long Covid patients is expected to grow considerably, to the extent that PHC will not be able to deal with this condition, considering the current human and financial resources available [72, 77, 78]. Given this scenario, there is an evident need for timely planning and allocation of necessary resources for care focused on patients with long Covid, as well as for preventing the worsening of their symptoms.

The main strengths of the present study are related to the considerable number of participants; the definition of a time limit for the characterisation of long Covid; the assessment of a wide range of symptoms related to long Covid, grouped according to body systems; the comparison of long Covid prevalence according to the exposure/severity factor in the acute phase of Covid‐19; and the identification of socio‐demographic, health and treatment variables associated with long Covid. However, there are several limitations to this study, namely the observational and cross‐sectional nature of the study, making it impossible to individually assess the duration of symptoms; the quality and reliability of information in the databases of the Covid‐19 notification systems and of the interviews carried out; and the use of self‐reported information, making it subject to recall bias, considering that the interviews were conducted 12 months after the occurrence of Covid‐19. Future research utilising larger and more varied cohorts, in conjunction with longitudinal studies, would enhance the robustness of our findings. Besides, this study relied solely on self‐reported data and lacked validation of the disease by the attending physician, as well as corroboration through electronic health records.

In addition, all participants were initially diagnosed with Covid‐19 before June 2021, a period during which either the wild‐type or alpha variant was predominant and before the widespread availability of Covid‐19 vaccinations [30]. The Alpha variant carries a higher risk of hospitalisation and severe Covid‐19, particularly among younger individuals [1]. This sample limitation diminishes the generalisability of results when applied to more recent variants. Lastly, data about other associated factors of long Covid‐19 (e.g., SARS‐CoV‐2 variants; previous vaccination against Covid‐19) is not available, resulting in a restricted number of variables that could be regarded as possible risk factors.

## 5 Conclusions

This study demonstrated a significant prevalence of long Covid among adults living in the State of Paraná and a wide variation in prevalence according to the severity of acute Covid‐19 infection. The long‐term effects of the disease represent an extremely important area that should be the focus of new research. Therefore, it is essential to direct efforts to better understand these persistent consequences across the population, to develop comprehensive strategies for prevention, rehabilitation and public health actions. Additional research is needed to describe the natural history of long Covid and characterise clusters of symptoms, their pathophysiology and clinical outcomes. More research is also needed to understand the health and social impacts of these persistent symptoms, to support patients living with long‐term sequelae and to develop targeted treatments. This study also suggests some important clinical implications. Early screening for long Covid should be prioritised in older female adults with multiple acute symptomatologic manifestations of Covid‐19, particularly those with multimorbidity. Healthcare providers should implement comprehensive management plans for long Covid, such as professional staff training programs for preventive interventions. Furthermore, it is necessary to stimulate social support networks through awareness and health education for the population, especially those who live in regions at increased risk for long Covid and with limited access to public health services.

## Author Contributions

**Eduardo Rocha Covre:** conceptualisation, writing – original draft, methodology, writing – review and editing, data curation, formal analysis, visualisation, investigation. **Carlos Laranjeira:** project administration, conceptualisation, methodology, formal analysis, writing – original draft, writing – review and editing, visualisation, funding acquisition. **Lígia Carreira:** writing – review and editing. **Carla Franciele Höring:** software, formal analysis. **Herbert Leopoldo de Freitas Góes:** writing – review and editing. **Vanessa Denardi Antoniassi Baldissera:** writing – review and editing. **Priscila Garcia Marques:** writing – review and editing. **Viviane Camboin Meireles:** writing – review and editing. **Maria Fernanda do Prado Tostes:** writing – review and editing. **Rosana Rosseto de Oliveira:** investigation, methodology. **Marcelle Paiano:** investigation, methodology. **Rosella Santoro Ageno:** writing – review and editing. **Márcia Moroskoski:** investigation, methodology. **Jesús Puente Alcaraz:** writing – review and editing. **João Nickenig Vissoci:** investigation, methodology. **Luiz Augusto Facchini:** investigation, methodology. **Maria Aparecida Salci:** conceptualisation, writing – review and editing, funding acquisition, supervision.

## Conflicts of Interest

The authors declare no conflicts of interest.

## Data Availability

The raw data supporting the conclusions of this article will be made available by the authors upon request.
